# Gene Co-Expression Network Analysis Identifies Vitamin D-Associated Gene Modules in Adult Normal Rectal Epithelium Following Supplementation

**DOI:** 10.3389/fgene.2021.783970

**Published:** 2022-01-04

**Authors:** James P. Blackmur, Peter G. Vaughan-Shaw, Kevin Donnelly, Bradley T. Harris, Victoria Svinti, Anna-Maria Ochocka-Fox, Paz Freile, Marion Walker, Toby Gurran, Stuart Reid, Colin A. Semple, Farhat V. N. Din, Maria Timofeeva, Malcolm G. Dunlop, Susan M. Farrington

**Affiliations:** ^1^ MRC Human Genetics Unit, Institute of Genetics and Cancer, University of Edinburgh, Edinburgh, United Kingdom; ^2^ Cancer Research UK Edinburgh Centre, Institute of Genetics and Cancer, University of Edinburgh, Edinburgh, United Kingdom; ^3^ Department of Public Health, Danish Institute for Advanced Study, University of Southern Denmark, Odense, Denmark

**Keywords:** colorectal cancer, vitamin D, vitamin D supplementation, gene correlation network, WGCNA (weighted gene co-expression network analysis)

## Abstract

Colorectal cancer (CRC) is a common, multifactorial disease. While observational studies have identified an association between lower vitamin D and higher CRC risk, supplementation trials have been inconclusive and the mechanisms by which vitamin D may modulate CRC risk are not well understood. We sought to perform a weighted gene co-expression network analysis (WGCNA) to identify modules present after vitamin D supplementation (when plasma vitamin D level was sufficient) which were absent before supplementation, and then to identify influential genes in those modules. The transcriptome from normal rectal mucosa biopsies of 49 individuals free from CRC were assessed before and after 12 weeks of 3200IU/day vitamin D (Fultium-D3) supplementation using paired-end total RNAseq. While the effects on expression patterns following vitamin D supplementation were subtle, *WGCNA* identified highly correlated genes forming gene modules. Four of the 17 modules identified in the post-vitamin D network were not preserved in the pre-vitamin D network, shedding new light on the biochemical impact of supplementation. These modules were enriched for GO terms related to the immune system, hormone metabolism, cell growth and RNA metabolism. Across the four treatment-associated modules, 51 hub genes were identified, with enrichment of 40 different transcription factor motifs in promoter regions of those genes, including VDR:RXR. Six of the hub genes were nominally differentially expressed in studies of vitamin D effects on adult normal mucosa organoids: *LCN2, HLA-C, AIF1L, PTPRU, PDE4B and IFI6.* By taking a gene-correlation network approach, we have described vitamin D induced changes to gene modules in normal human rectal epithelium *in vivo*, the target tissue from which CRC develops.

## Introduction

Colorectal cancer (CRC) is common, with over 40,000 incident cases and over 15,000 deaths associated with the disease per year in the United Kingdom (Cancer Research UK, 2019). In case-control and prospective cohort studies, higher plasma 25(OH)D level and higher dietary intake of vitamin D are associated with lower CRC risk (Jenab et al., 2010; Theodoratou et al., 2012; Theodoratou et al., 2014). Potential co-causality of CRC risk and vitamin D status (e.g. socioeconomic status, diet, physical exercise) and reverse causation (CRC or its treatment affecting serum vitamin D concentration) mean these observational studies may be confounded. Supplementation trials have been inconclusive, with randomised-controlled trials (RCTs) failing to show effect on CRC or adenoma (precursor lesion) incidence (Wactawski-Wende et al., 2006; Baron et al., 2015; Manson et al., 2019; Scragg, 2019). However, these supplementation studies are themselves confounded by short duration of follow up and vagaries in genetic, environmental, ethnic, dietary and ecological factors such as latitude, weather and sunlight exposure, with many participants in both control and intervention arms starting trials replete in vitamin D, and/or taking low-dose vitamin D supplementation. Whether vitamin D supplementation reduces risk of CRC therefore remains an open question. In addition, recent studies have suggested a beneficial effect for vitamin D supplementation on CRC mortality (Keum et al., 2019; Vaughan-Shaw et al., 2020).

The mechanisms by which vitamin D may modulate CRC risk and survival are not well understood (Alleyne et al., 2017). Potential mechanisms include induction of cell differentiation and apoptosis or inhibition of cell growth and proliferation (Lamprecht and Lipkin, 2003; Feldman et al., 2014). Understanding of vitamin D transcriptional responses in the colon and rectum has primarily come from studies in transformed cancer cell lines, which may not represent responses in normal healthy tissue (Mapes et al., 2014), hence it is advantageous and timely to prioritise human studies to explore potential mechanisms in the normal target tissue.

Gene co-expression networks are used to describe the pairwise relationships of a large number of gene expression variables (Zhang and Horvath, 2005). Genes related by high correlation coefficients are thought to be functionally related, members of the same pathway and/or co-regulated. Weighted gene co-expression network analysis (*WGCNA*) is one of the most widely used network methods, whereby gene correlations are raised by a soft-thresholding power to form a scale-free network. The advantage of this method is that all correlations are included for analysis. Methods that apply a hard-threshold (e.g. r > 0.7 being arbitrarily biologically relevant) lose connections below that threshold for further analysis (Zhang and Horvath, 2005). Highly connected genes within each module identified by *WGCNA* are defined as hub genes, with such genes thought to play key biological roles in that particular module or in regulation of a particular trait (Zhang and Horvath, 2005; Kogelman et al., 2014; Drag et al., 2017; Bakhtiarizadeh et al., 2018). By comparing differences in network structures (e.g. whether gene modules are preserved between conditions), it is possible to assess how groups of genes are perturbed by a certain condition (such as vitamin D supplementation) (Langfelder et al., 2011). This method has been successfully used to discover genes involved in endometriosis (Bakhtiarizadeh et al., 2018) and multiple cancer types (Sun et al., 2017; Zhang et al., 2018; Zhu et al., 2019).

In this study, we aimed to determine effects of vitamin D on normal rectal epithelium by assessing gene expression before and after 12 weeks of vitamin D supplementation in human subjects. We first assessed vitamin D effects on the expression of single genes, and then constructed a weighted correlation network to assess effects of vitamin D on groups of genes, and to identify hub genes in modules emerging after supplementation. We sought to functionally annotate genes and gene modules using Gene Ontology (GO) pathway analysis and to determine transcription factor binding sites common to hub genes in emergent modules. Finally, we sought to validate expression changes of hub genes in adult normal colorectal mucosa organoids treated with vitamin D. These organoid models are isolated from many of the vagaries in heterogeneity of the human population, yet maintain the genetic architecture and 3D-cell arrangement present in the parent tissue.

## Materials and Methods

### Human Vitamin D Supplementation Study

In the Scottish Vitamin D (SCOVID) study, rectal normal mucosa biopsies (via rigid sigmoidoscopy) and blood were collected after informed consent from a cohort of human subjects free from colorectal cancer (n = 50, 49 whose samples passed QC). Demographic information is provided in Table 1, while the study protocol has been described elsewhere (Vaughan-Shaw et al., 2021). The study had approval from NHS Research Ethics Committee (REC No 13/SS/0248) and local Research and Development Committee (R&D Project ID 2014/0058). Participants received 3200IU daily oral vitamin D (Fultium-D3), with resampling at 12 weeks. Biopsy samples were stored immediately in RNA Later (Invitrogen) and kept for 48 h at 4°C before RNA extraction.

**TABLE 1:** Demographic and clinical information of SCOVID study participants.

Factor		
Age	Median years (IQR)	66 (58–72)
Gender	F/M	23/26
BMI	Median kg/m2 (IQR)	26.21 (23.66–31.64)
Current CRC	N/Y	49/0
Past History CRC	N/Y	31/18
Pre-supplementation		
Plasma 25(OH)D	Median nmol/l (IQR)	86 (23–54)
	*n* < 25 nmol/l	15
	*n* 25–50 nmol/l	20
	*n* > 50 nmol/l	14
Post-supplementation		
Plasma 25(OH)D	Median nmol/l (IQR)	89 (71–109)
	*n* < 25 nmol/l	0
	*n* 25–50 nmol/l	1
	*n* > 50 nmol/l	48

Plasma 25(OH)D was assayed from blood by mass spectrometry. Plasma extracted from blood taken in lithium heparin tubes was immediately frozen at −40°C and subsequently submitted to the Clinical Biochemistry department, Glasgow Royal Infirmary, United Kingdom for measurement of 25(OH)D.

To extract RNA, human biopsy samples transferred to 2 ml Eppendorf tubes and homogenised in Trizol. RNA was then extracted by Ribopure Kit (Invitrogen) according to the manufacturer’s protocol. RNA samples from 49 subjects passed QC and were submitted to the Edinburgh Genomics facility, with sequencing on the Illumina HiSeq 2,500 in “rapid mode” with 150 bp paired-end reads as described in Supp Methods.

### Analysis

Transcript quantification from RNAseq was conducted using *Salmon* v0.11 (Patro et al., 2017) using Ensembl version GRCh38, March 2017, Ensembl 88. Gene level counts were generated by R packages *txiimport* (Soneson et al., 2015) and annotated using *biomaRt* (Durinck et al., 2005; Durinck et al., 2009). Expression was normalised using a TMM algorithm based on gene expression thresholds of >0.1 Transcripts Per Million (TPM) and ≥6 reads in ≥20% of samples. Trimmed mean of M-values (TMM) between-sample normalisation was applied to the counts, as per the GTEx (v7) protocol. Effects of vitamin D on gene expression were assessed using *edgeR v3.32.1* (Robinson et al., 2010)*.* Following dispersion estimation, a quasi-likelihood negative binomial generalized log-linear model was fitted to the count data. Differential expression was assessed by quasi-likelihood F-test, with the parent-tissue anonymous identifier included as a covariate in the model as per a paired-design. Significance was determined as FDR corrected *p* value < 0.05. GO pathway analysis was carried out by clusterProfiler (Yu et al., 2012).

Normalised counts were first log transformed before proceeding to correlation analysis by *WGCNA*. To minimise the biological noise from genes not functionally related to vitamin D and to limit the dataset for computational analysis, the top 25% most variable genes after vitamin D supplementation (determined by logFC) were taken forward for analysis. *WGCNA* analysis is sensitive to the presence of outliers (Horvath, 2011), therefore samples with a standardized connectivity score of less than −5 were removed. The goodSamplesGenes function was then used to remove samples and genes with missing entries (more than 50% missing entries) and genes with zero variance.

Vitamin D supplementation achieved a 25(OH)D concentration which might be termed “healthy” whereas the pre-supplementation samples were depleted in 25(OH)D, and could be termed the “disease” state. Based on the assumption that modules present after vitamin D supplementation but not present before would be of interest in discerning vitamin D activity, the post-vitamin D samples were considered as the reference set for module derivation. Module preservation analysis was then undertaken in the pre-vitamin D samples. Using *WGCNA*, we created a signed weighted gene co-expression network based on normal gene expression data. A weighted network was created from the pairwise biweight midcorrelation coefficients between genes using the *blockwiseModules* function, with module merge cut height of 0.25 and a minimum module size of 30 genes (Song et al., 2012; Bakhtiarizadeh et al., 2018). A weighted adjacency matrix was formed by raising correlations to the power of 7, which was chosen using the scale-free topology criterion (Zhang and Horvath, 2005; Horvath, 2011). The relationship between the power (*β*) and *R* ^2^ for a scale-free network is demonstrated along with sample dendrograms and their trait relationships in Supplementary Figure S1.

Age, gender and body mass index (BMI) have been reported to be associated with vitamin D concentration (Lagunova et al., 2009; Muscogiuri et al., 2019) and hence to assess association of those traits with identified gene modules, we assessed trait correlations to module eigenvectors (first principal component of each module). Each module eigenvector represents the expression profiles of all genes within that module.

To assess the preservation of post-vitamin D network modules in the pre-vitamin D dataset, the *modulePreservation* function in the *WGCNA* package was applied. We then applied Zsummary and medianRank (with 200 permutations) to detect module preservation. A module was considered as non-preserved if it had Zsummary<5 or medianRank≥8 (Langfelder et al., 2011).

To identify hub genes, intramodular connectivity (kIM) and module membership (kME) measures were used. Intramodular connectivity measures the degree of co-expression of a given gene with respect to the genes of a particular module. This was determined for both post- and pre-supplementation networks from the respective matrices of log transformed normalised counts using the *intramodularConnectivity.fromExpr* function (using pairwise biweight midcorrelation coefficients, power 7, a signed network and using the module labels identified above). Module membership was determined similarly by the *signedKME* function, which determines the correlation between the expression profile of a gene and the module eigengene (first principal component of a particular module). Genes were kME ≥0.7 or kIM ≥0.7 were considered as hub genes to the respective module (Horvath, 2011).

### Validation of Gene Modules in the STRING Dataset

We sought to validate gene modules identified by *WGCNA* in the STRING (Search Tool for the Retrieval of Interacting Genes/Proteins) curated database of protein-protein interactions (https://string-db.org/). The STRING database collects, scores and integrates publicly available sources of protein–protein interaction information, and can be used to assess if groups of genes identified by the user are enriched for protein-protein interactions in those sources (Szklarczyk et al., 2019). HGNC gene names within each module were uploaded to the STRING web-browser, and interactions assessed across all seven of the STRING “interaction sources”.

### Investigation of Common Transcription Factor Binding Sites

We were interested to assess if hub genes in non-preserved modules may be regulated by common transcription factors. As per Bakhtiarizadeh et al. (2018), the “TRANSFAC_and_JASPAR_PWMs” section of the Enrichr tool (Chen et al., 2013) (https://amp.pharm.mssm.edu/Enrichr/) was applied to determine common transcription factor binding sites in promoter regions of such genes.

### Validation of Hub Gene Expression Changes in Adult Normal Colorectal Organoids

Fernandez-Barral et al. (2020) undertook differential expression analysis of adult normal mucosa organoids (FB-ANMO) derived from six individuals treated for 96 h with 100 nM calcitriol or 1% ethanol. Normalised counts and anonymised meta-data were downloaded from GEO (GSE100785; https://www.ncbi.nlm.nih.gov/geo/query/acc.cgi?acc = GSE100785). The same edgeR pipeline as described above was then used to determine differentially expressed genes in the FB-ANMO. Gene set testing was carried out using the *geneSetTest* function in *limma* (Ritchie et al., 2015).

## Results

### No Genes Were Differentially Expressed at a Genome-Wide Level Following Vitamin D Supplementation

Fultium-D3 supplementation increased plasma 25(OH)D in all study participants [mean (SD) nmol/l baseline 39.4 (20.4), 12 weeks 92.2 (27.0), *p* < 0.001; mean increase 52.8 (28.3)]. 21,650 genes passed expression filters and were taken forward for differential expression analysis. 2,492 genes were nominally differentially expressed (*p* < 0.05), but none remained significant after genome-wide FDR correction (FDR *p* < 0.05, Supplementary Table S1). 150 GO Biological Process terms were enriched in nominally differentially expressed genes including “protein-containing complex localization”, “DNA conformation change”, and “RNA splicing” (Supplementary Table S2).

### Gene Modules Identified by *WGCNA* After Vitamin D Supplementation

Having identified no genes differentially expressed at a genome-wide significance level, we were interested to examine if vitamin D changes occurred across groups of genes by network connectivity analysis. From the total pool of 21,650 genes, the top 25% (5,412) genes by logFC (irrespective of direction of effect) were taken forward for further analysis. This limited analysis to the genes potentially most responsive to vitamin D supplementation for computational reasons. No samples had outlying standardized connectivity score, and no genes were lost by the *goodSamplesGenes* function.

**FIGURE 1:**
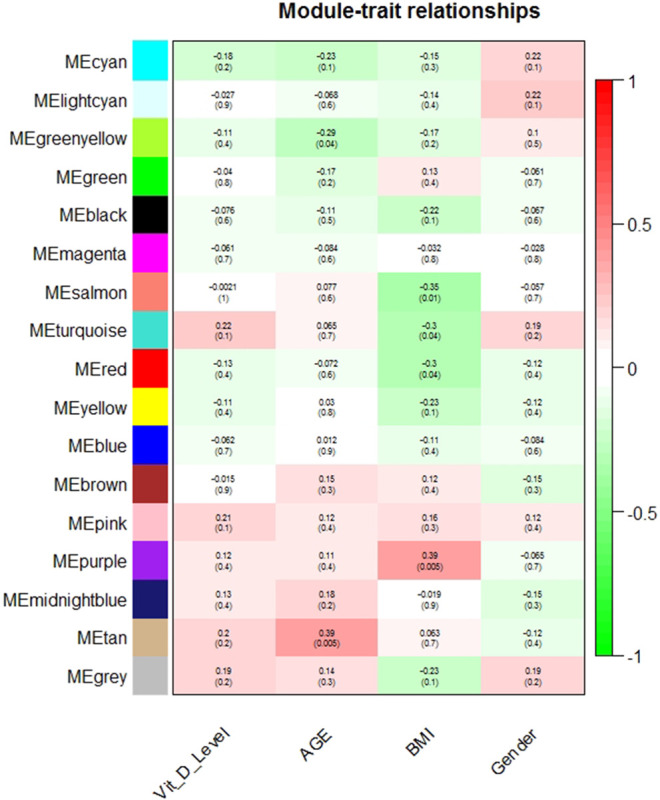
Module-trait relationships in the post-vitamin D network. Pearson correlation of module eigenvector (first principal component) and trait (along with nominal *p*-value).

17 modules were identified in the post-vitamin D network including the unclassified module (grey). No module eigenvector was correlated with plasma 25(OH)D or gender (Figure 1, Supplementary Table S3). The tan and greenyellow module eigenvectors were nominally significantly correlated with age, though did not remain significantly correlated after correction for multiple testing (r = 0.39, *p* = 0.005, FDR *p* = 0.09; r = -0.29, *p* = 0.04, FDR *p* = 0.38 respectively). The purple, salmon, red and turquoise module eigenvectors were nominally correlated with BMI, however again were not significant after accounting for multiple testing (r = 0.39, *p* = 0.005, FDR *p* = 0.09; r = -0.35, *p* = 0.01, FDR *p* = 0.13; r = -0.30, *p* = 0.04, FDR *p* = 0.16; r = -0.30, *p* = 0.04, FDR *p* = 0.16 respectively).

### Identification of Treatment-Associated Modules From the Pre-Vitamin D Transcriptome

**FIGURE 2:**
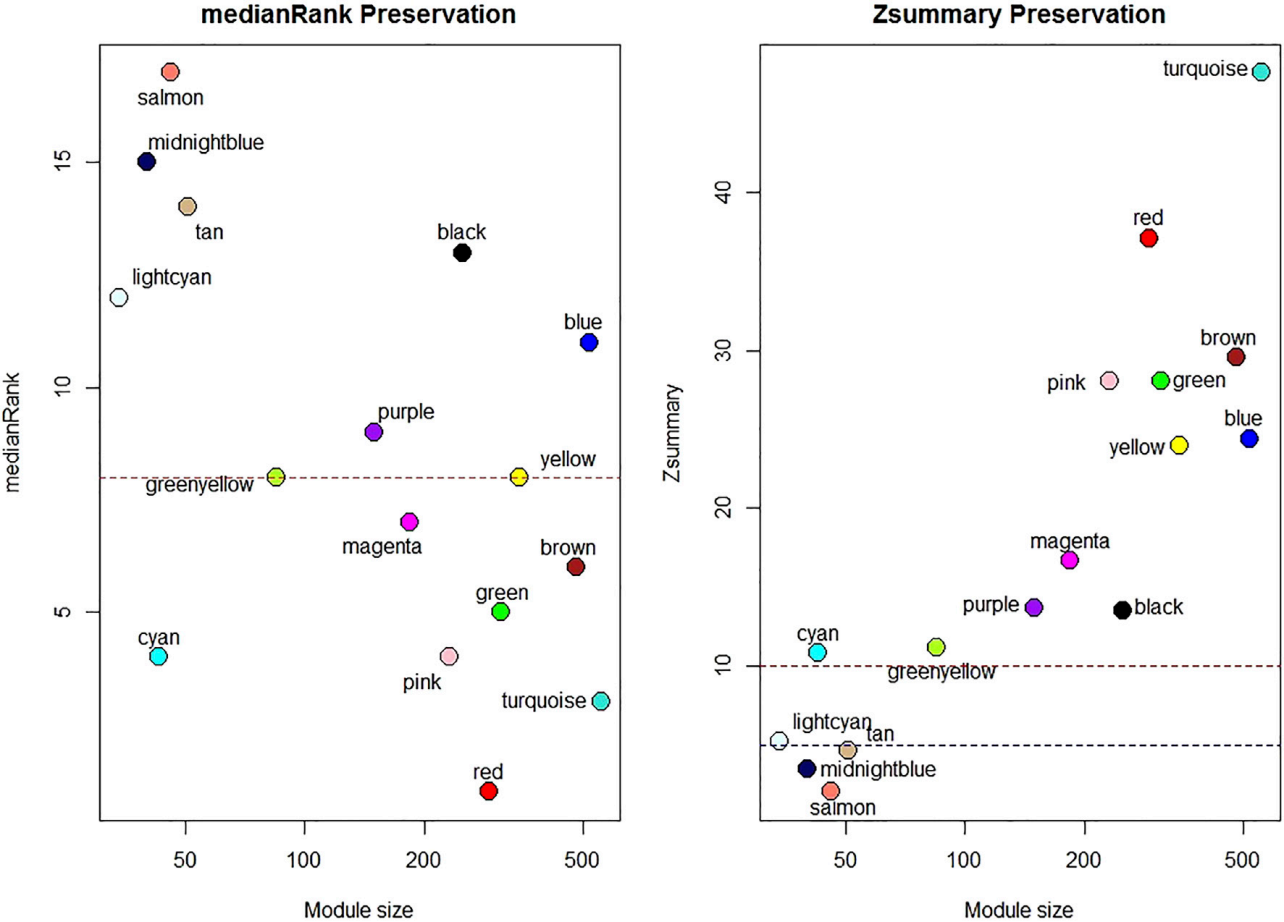
Median rank and Z-summary statistics for preservation of modules from the post-vitamin D network in the pre-vitamin D network. Z-statistic >10 strong evidence of preservation, 5–10 moderate evidence of preservation, 2–5 weak evidence of preservation and <2 no evidence of preservation (Langfelder et al., 2011).

One sample with outlying standardized connectivity score was removed from the pre-vitamin D network prior to module preservation analysis. 12 modules from the post-vitamin D network were strongly preserved in the pre-vitamin D network (defined as Zsummary statistic >10), i.e. were not associated with vitamin D treatment, while four modules from the post-vitamin D network were not preserved in the pre-vitamin D network, and hence were considered to be treatment-associated modules (Zsummary statistic <5 and median rank >8 for salmon, midnightblue and tan modules. Lightcyan module Zsummary statistic 5.2, Zdensity. pres 4.1 and Zconnectivity 6.3 with median rank 12). Module preservation statistics are described in Supplementary Table S4 and their distribution in Figure 2. GO terms for each module are described in Table 2 and Supplementary Table S5.

**TABLE 2:** Select GO biological process terms in each of the modules present in the post-vitamin D network, with associated statistics for preservation in the pre-vitamin D network. The top three GO biological process terms for each module (determined by FDR *p*-value) are shown. Where highly similar terms related to overlapping genes existed (e.g., purine ribonucleoside binding and purine nucleoside binding) only one term is shown.

Module	Module size	Z summary	Median rank	N GO terms	GO description
Salmon	46	2.0	17	9	Defense response to virus; RNA helicase; GTP binding;
Midnightblue	40	3.47	15	158	viral mRNA export from host cell nucleus; RNA secondary structure unwinding; negative regulation of DNA damage checkpoint
Tan	51	4.60	14	5	positive regulation of hormone metabolic process; sphingolipid mediated signaling pathway; positive regulation of nuclear division
Lightcyan	34	5.21	12	49	adaptive immune response based on somatic recombination of immune receptors built from immunoglobulin superfamily domains; regulation of leukocyte mediated cytotoxicity
Cyan	43	10.83	4	141	ribosome biogenesis; ncRNA processing; nucleocytoplasmic transport
Greenyellow	85	11.19	8	202	homophilic cell adhesion via plasma membrane adhesion molecules; extracellular matrix organization; smoothened signaling pathway
Black	249	13.48	13	603	extracellular matrix organization; muscle contraction; axonogenesis
Purple	150	13.65	9	112	RNA splicing, via transesterification reactions; cilium organization; RNA transport
Magenta	184	16.64	7	166	positive regulation of viral release from host cell; vacuolar transport; endosome organization
Yellow	348	23.94	8	783	lymphocyte differentiation; regulation of T cell activation; positive regulation of leukocyte cell-cell adhesion
Blue	521	24.32	11	94	peptidyl-lysine modification; TORC1 signaling; histone modification
Pink	231	28.05	4	100	alcohol metabolic process; cellular response to extracellular stimulus; macroautophagy
Green	311	28.05	5	466	oxidative phosphorylation; cellular respiration; mitochondrial translation
Brown	481	29.54	6	5	apoptotic process involved in morphogenesis
Red	290	37.08	1	375	muscle tissue development; extracellular matrix organization; multicellular organismal signaling
Turquoise	556	47.57	3	445	leukocyte cell-cell adhesion; regulation of T cell activation; lymphocyte proliferation

As a means of testing if *WGCNA* had identified gene modules which had also been found to be interacting in other datasets, we next sought to test if these modules were enriched in the STRING curated database of protein-protein interactions (PPI). Three of the four modules which were not preserved in the pre-vitamin D network were enriched for protein-protein interactions (Table 3). In addition 11 of the 12 preserved modules were enriched for protein-protein interactions (Supplementary Table S6).

**TABLE 3:** Protein-protein interaction enrichment of genes in non-preserved modules identified in the STRING database. FDR correction for 16 modules tested (grey unclassified module excluded).

Module	N genes in string database	N genes in module	Number of connections	PPI enrichment *p* value	PPI enrichment FDR *p* value
Salmon	19	46	13	1.12e-05	1.28E-05
Midnightblue	22	40	12	5.14e-07	6.85E-07
Tan	38	47	6	0.093	0.099
Lightcyan	24	28	29	<1.0e-16	<2.67E-16

### Identification of Hub Genes in Treatment-Associated Modules

The genes with the highest degree of connectivity within a particular module are considered as hub genes. We were interested to identify hub genes in treatment-associated (non-preserved) modules, and in particular those genes which gained or lost hub-status. Hub genes were defined as those with kME ≥0.7 or with kIM≥0.7. By this definition, eight hub genes were identified in the salmon module, 13 in the midnightblue, 21 in the tan and nine in the lightcyan module (Supplementary Table S7).

### Enrichment of Transcription Factor Binding Sites in Hub Gene Promoter Regions

Given hub genes are thought to be functionally important within their respective modules, we next sought to identify transcription factors common to hub genes in the non-preserved modules. A total of 40 different transcription factor motifs were enriched in promoter regions of hub genes in treatment-associated modules (Supplementary Table S8). Each of the four treatment-associated modules included hub genes which contained either VDR:RXR or RXR binding motifs in their respective promoter regions.

### Crossover With Differentially Expressed Genes in NM Organoids

Finally, we reviewed differential expression of hub genes in a study of adult normal mucosa organoids treated with vitamin D. 15 of the 51 hub genes in treatment-associated modules identified above were present in the FB-ANMO dataset after gene filters. Those 15 genes were significantly downregulated on gene set testing in FB-ANMO (*p* = 0.02). Six of the 15 hub genes were also nominally differentially expressed in FB-ANMO, with five remaining significant after correction for multiple testing (Genome-wide FDR <0.05, Table 4, Supplementary Table S9). *WGCNA* is not recommended on datasets with fewer than 15 samples (Langfelder and Horvath, 2017), and hence we did not proceed to network analysis in this organoid dataset (6 samples per condition).

**TABLE 4:** Hub genes in non-preserved modules which are also differentially expressed in adult normal mucosa organoids from re-analysis of Fernandez-Barral et al. (FB-ANMO).

Gene	Module	logFC SCOVID	Pvalue	kME post	kME pre	kIM post	kIM pre	logFC organoid	Pvalue organoid	FDR organoid
*LCN2*	Tan	0.20	1.17E-01	0.47	0.57	0.75	0.85	-0.62	1.51E-04	2.27E-03
*AIF1L*	Tan	0.12	4.79E-02	0.44	0.40	0.58	0.71	-0.70	1.38E-03	1.03E-02
*HLA-C*	Lightcyan	0.14	4.30E-01	0.82	0.80	0.86	0.89	0.22	2.92E-03	1.31E-02
*PTPRU*	Tan	0.21	8.56E-02	0.64	0.61	0.94	0.92	-0.28	3.49E-03	1.31E-02
*PDE4B*	Tan	0.12	2.39E-01	0.50	0.63	0.72	0.92	0.88	8.90E-03	2.67E-02
*IFI6*	Salmon	0.17	1.60E-01	0.34	0.55	0.33	0.74	-0.33	3.38E-02	8.45E-02

## Discussion

In this study, we have demonstrated that while vitamin D supplementation does not affect the expression of any single gene at a genome-wide level of significance, it does induce changes in the inter-correlated expression patterns across genes, reflected in modules. 16 gene modules were identified after vitamin D supplementation (a state which reflects those individuals having a sufficient level of vitamin D for health). 14 of the 16 identified modules were also noted to be significantly enriched for protein-protein interactions in the STRING curated database, suggesting that *WGCNA* is able to identify modules which may have functional relevance.

Setting these results in context, other studies have similarly found no or few differentially expressed single genes following vitamin D supplementation (Hossein-nezhad et al., 2013; Munro, 2016; Pasing et al., 2017), however pathway analyses have identified effects on fatty acid metabolism and PPAR signaling (Munro, 2016), MAPK signaling, NF-kappa B signaling, T cell receptor signaling and prostate cancer (Hossein-nezhad et al., 2013). No study has previously investigated effects of vitamin D supplementation by the gene-correlation network approach. We were particularly interested to identify four treatment-associated modules that were not observed before vitamin D supplementation (a state of vitamin D insufficiency). Functional terms enriched in those modules included multiple GO terms related to the immune system, along with terms related to hormone metabolism, cell growth and RNA metabolism. Enrichment of GO terms associated with immunity is of particular note given the general interest in vitamin D and the immune system, in particular with risk of conditions such as multiple sclerosis and inflammatory bowel disease being associated with lower serum vitamin D (Munger et al., 2006; Limketkai et al., 2017; Fletcher et al., 2019). Vitamin D has also been postulated to favourably benefit the immune response in severe acute respiratory syndrome coronavirus 2 (SARS-CoV-2) (Martineau and Forouhi, 2020). Mechanistically, an excess of VDR binding variants identified by ChIP-exo has been reported to overlap with genomic variants associated with autoimmune disorders such as inflammatory bowel disease, Crohn’s disease and rheumatoid arthritis (Gallone et al., 2017).

Hub genes represent the most connected genes within a module, and are thought to be functionally important. Interestingly each of the non-preserved modules contained hub genes which contained VDR:RXR or RXR motifs in their respective promoter regions. Vitamin D signaling occurs principally following the binding of the active form of vitamin D, 1,25(OH)_2_D, to the Vitamin D Receptor (VDR) (Kliewer et al., 1992). VDR forms a heterodimer complex with Retinoid X Receptor (RXR) to bind DNA via VDR-responsive elements (VDRE) largely characterized by the VDR-RXR motif (Kliewer et al., 1992; Zhang et al., 2011). While the presence of a motif does not indicate actual transcription factor binding *in-vivo*, it does add further support to the notion that these genes are central to the vitamin D response in normal rectal epithelium.

Finally, when the list of hub-genes in non-preserved modules was cross-referenced with a separate study of vitamin D response in adult normal mucosa organoids, a number of interesting candidate genes were identified including *LCN2*, *HLA-C* and *IFI6*. *LCN2,* found to have a RXR motif in its promoter region, is expressed by macrophages and epithelia in response to inflammation (Dahl et al., 2018) and inhibition of *LCN2-*modulated NF-kB pathway activation by vitamin D has been noted to promote cisplatin sensitivity of oral squamous cell carcinomas (Huang et al., 2019). *LCN2* acts in a bacteriostatic fashion (Dahl et al., 2018), which is noteworthy given the potential role of the gut microbiota in development of CRC (Saus et al., 2019). Human leukocyte antigens have been reported to be a major target of vitamin D physiological activity (Carlberg, 2019), with *HLA-C* being differentially expressed in peripheral blood mononuclear cells (PBMCs) following vitamin D supplementation of adult humans (Neme et al., 2019). Interferon alpha-inducible protein 6 (*IFI6*), also known as *G1P3* has been shown to contribute to hyperplasia, tamoxifen resistance and poor outcomes in breast cancer (Cheriyath et al., 2012). It was one of the top differentially expressed genes (log FC -3.04) following vitamin D treatment of airway smooth muscle cells derived from individuals following a fatal asthma episode (Himes et al., 2015).

It is worthy of note that no module eigenvector was correlated with plasma 25(OH)D. The relationships between vitamin D and gene expression may not be linear, and instead sigmoidal or U-shaped relationships may exist (Mizunashi et al., 1995; Ross et al., 2011; Macdonald et al., 2018). Assessing the relationships between plasma 25(OH)D and gene modules may therefore fail to show effects in individuals sufficient in vitamin D, as was the case following vitamin D supplementation in this study. In addition, plasma vitamin D may not be an accurate measure of vitamin D in the target tissue of interest (in this case the rectal epithelium), with previous studies reporting a marked discrepancy between serum and tissue concentrations (Martinaityte et al., 2017).

This study represents a novel approach to assessing vitamin D effects. Unlike many of the large randomised-controlled trials of vitamin D effects (Wactawski-Wende et al., 2006; Baron et al., 2015; Manson et al., 2019; Scragg, 2019), the majority of participants were deficient in vitamin D at the start of the study period (*n* = 39 baseline plasma 25(OH)D < 50 nmol/l). The effects of vitamin D were also assessed directly in the target tissue of interest, the rectum, as opposed to assessing effects in blood which may have been technically easier to sample. This study is larger than many of the published studies assessing effects of vitamin D supplementation on gene expression (Hossein-nezhad and Holick, 2013; Gerke et al., 2014; Ryynänen et al., 2014; Protiva et al., 2016). Limitations of this study have been discussed elsewhere (Vaughan-Shaw et al., 2021). This study may have been too small to achieve sufficient power to assess individual gene significance. Individuals taking part in the study were not selected on the basis of initial plasma 25(OH)D; supplementation may have a sigmoidal or U-shaped relationship with gene expression (Mizunashi et al., 1995; Ross et al., 2011; Macdonald et al., 2018) and hence failing to select participants based on initial 25(OH)D could blunt the observed effect of supplementation. Finally sampling after 12 weeks of supplementation may not adequately capture early or later gene expression changes, however more frequent or delayed sampling would provide additional practical and ethical challenges.

## Summary

By taking a gene-correlation network approach, we have described vitamin D-induced changes to groups of genes in normal human rectal epithelium. By reviewing treatment-associated modules before and after vitamin D supplementation, we have identified hub genes which may play a key role in modulating vitamin D actions in normal rectal epithelium. This provides novel understanding of the mechanisms by which vitamin D may have beneficial effects on CRC risk and survival.

## Data Availability

The datasets presented in this study can be found in online repositories. The names of the repository/repositories and accession number(s) can be found below: https://www.ncbi.nlm.nih.gov/, GSE157982.
