# Supplementary figures and images for: Gene Co-Expression Network Analysis Identifies Vitamin D-Associated Gene Modules in Adult Normal Rectal Epithelium Following Supplementation
Source: Front Genet. 2022 Jan 4;12:783970. doi: 10.3389/fgene.2021.783970 (PMC8790603; doi:10.3389/fgene.2021.783970)

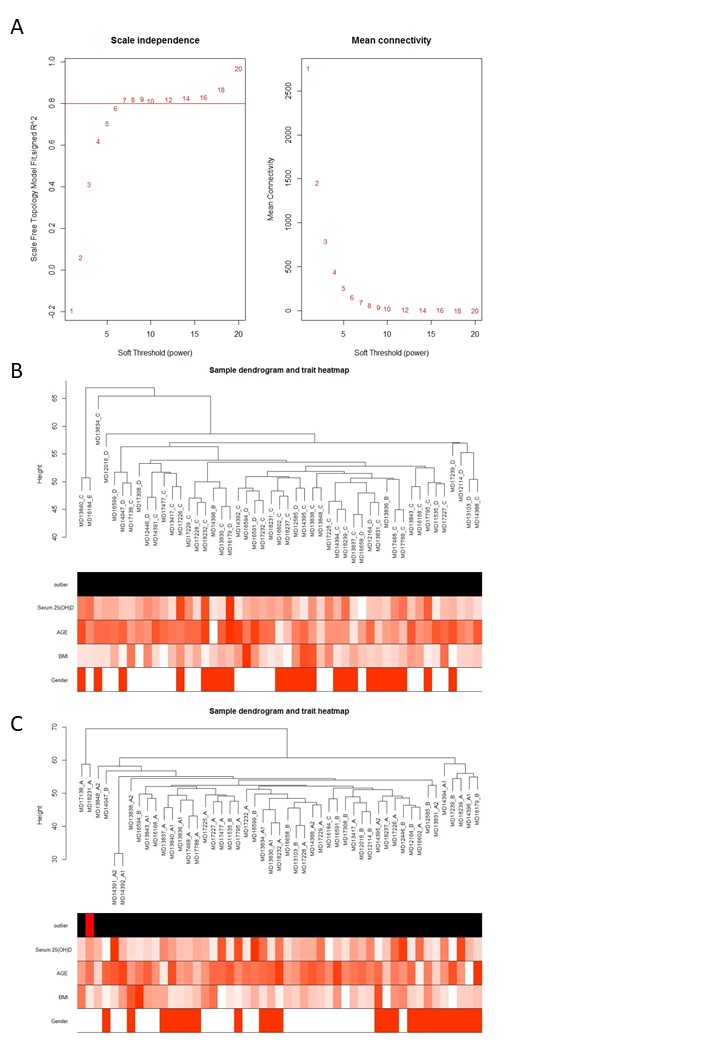

Supplement: Supplementary file 1 [file Image1.JPEG]
