# Supplementary material for: Gene Co-Expression Network Analysis Identifies Vitamin D-Associated Gene Modules in Adult Normal Rectal Epithelium Following Supplementation
Source: Front Genet. 2022 Jan 4;12:783970. doi: 10.3389/fgene.2021.783970 (PMC8790603; doi:10.3389/fgene.2021.783970)
Supplement: Supplementary file 2 [file DataSheet2.docx]

Supplementary Methods

RNA samples were subjected to 150bp paired-end total RNA-seq (155M reads) in a single batch. RNA integrity and yield were quantified using the 2100 Bioanalyzer. Extracted RNA was reverse transcribed using Moloney Murine Leukemia Virus reverse transcriptase (Promega) and random primers (Promega) at 37^o^C for 30 minutes and 95^o^C for 5 minutes. RNA samples were submitted to the Edinburgh Genomics sequencing facility, where QC, ribosomal-depletion, strand-aware library preparation and Illumina adapter ligation was performed. Ribosomal RNA was depleted using the New England Biolabs NEBNext rRNA Depletion Kit according to the manufacturer’s protocol. Samples were sequenced on the Illumina HiSeq 2500 in “rapid mode”.

Transcript quantification from RNAseq was conducted using *Salmon* v0.11 (1) using Ensembl version GRCh38, March 2017, Ensembl 88. In brief, the Ensembl (cDNA and ncRNA) and reference FASTA were concatenated, the ‘decoys.txt’ was prepared from GRCh38 and the Salmon index run on the concatenated FASTA file. Paired reads 1 and read 2 fastq files respectively were then concatenated for each sample, and Salmon run using auto-detect strandedness and ‘validateMappings’ flag. The percentage of mapped reads varied between 41 and 64%. Gene level counts were generated by R packages *txiimport (2)* and *biomaRt* (3, 4).

References

1. Patro R, Duggal G, Love MI, Irizarry RA, Kingsford C. Salmon provides fast and bias-aware quantification of transcript expression. Nat Methods. 2017;14(4):417-9.

2. Soneson C, Love MI, Robinson MD. Differential analyses for RNA-seq: transcript-level estimates improve gene-level inferences. F1000Res. 2015;4:1521.

3. Durinck S, Spellman PT, Birney E, Huber W. Mapping identifiers for the integration of genomic datasets with the R/Bioconductor package biomaRt. Nat Protoc. 2009;4(8):1184-91.

4. Durinck S, Moreau Y, Kasprzyk A, Davis S, De Moor B, Brazma A, et al. BioMart and Bioconductor: a powerful link between biological databases and microarray data analysis. Bioinformatics. 2005;21(16):3439-40.
